# Nanodelivery of a functional membrane receptor to manipulate cellular phenotype

**DOI:** 10.1038/s41598-018-21863-3

**Published:** 2018-02-23

**Authors:** Tommaso Patriarchi, Ao Shen, Wei He, Mo Baikoghli, R. Holland Cheng, Yang K. Xiang, Matthew A. Coleman, Lin Tian

**Affiliations:** 1 University of California Davis, School of Medicine, Department of Biochemistry and Molecular Medicine, Davis, California USA; 2 University of California Davis, School of Medicine, Department of Pharmacology, Davis, California USA; 3 Lawrence Livermore National Laboratory, Livermore, California USA; 4 University of California Davis, Department of Molecular and Cellular Biology, California, USA; 5 VA Northern California Health care system, Mather, California USA; 6 University of California Davis School of Medicine, Radiation Oncology, Sacramento, California USA

## Abstract

Modification of membrane receptor makeup is one of the most efficient ways to control input-output signals but is usually achieved by expressing DNA or RNA-encoded proteins or by using other genome-editing methods, which can be technically challenging and produce unwanted side effects. Here we develop and validate a nanodelivery approach to transfer *in vitro* synthesized, functional membrane receptors into the plasma membrane of living cells. Using β_2_-adrenergic receptor (β_2_AR), a prototypical G-protein coupled receptor, as an example, we demonstrated efficient incorporation of a full-length β_2_AR into a variety of mammalian cells, which imparts pharmacologic control over cellular signaling and affects cellular phenotype in an *ex-vivo* wound-healing model. Our approach for nanodelivery of functional membrane receptors expands the current toolkit for DNA and RNA-free manipulation of cellular function. We expect this approach to be readily applicable to the synthesis and nanodelivery of other types of GPCRs and membrane receptors, opening new doors for therapeutic development at the intersection between synthetic biology and nanomedicine.

## Introduction

Membrane proteins play important roles in all aspects of cellular signaling and are the interface through which a cell responds to extracellular cues. One of the most important subsets of the cellular membrane protein makeup are G-protein–coupled receptors (GPCRs), which sense a variety of external cues to orchestrate a broad range of cognitive and physiological responses and are targets for approximately 30% of all clinically-approved drugs^1^. Therefore, modulation of GPCRs is a powerful means to manipulate cellular signaling and phenotype, enabling a broad spectrum of research and clinical applications. Unfortunately, expression of functional membrane proteins can only been achieved through the introduction of DNA or RNA-based genetic material into cells, which can be technically challenging in primary cells and offers poor control over the amounts of protein being produced^2^.

To obtain pure and soluble GPCRs while maintaining proper folding and transitions between conformational states, individual receptor molecules need to be in a native-like environment, which can be provided with the use nanolipoprotein particles (NLPs), also known as nanodiscs^3,4^. Nanodiscs hold great promise as a platform for efficient cellular or *in vivo* delivery of membrane-associated molecules, due to their highly bio-compatible properties (e.g. stability, lack of toxicity, biodistribution)^5^. However, their use has been limited to the delivery of drugs^6^, phospholipids^7^ and personalized cancer vaccines^8^, while nanodelivery of functional membrane receptors has not been reported.

Traditionally, nanodisc-solubilized GPCRs have been produced using cell-based methods that require expression in cell hosts followed by detergent extraction and nanodisc reconsitution, which is time-consuming and labor intensive^3,9^. Cell-free synthesis provides a viable alternative for large-scale production of membrane proteins and is emerging as a competitive choice due to its increasing production yields (up to mg/ml reaction amounts in the most optimized systems) and to its lower expression costs^9–15^, especially with the adoption of alternative energy regeneration systems for protein synthesis^16^.

Here, we developed a platform integrating cell-free production and nanodelivery of functional GPCRs to the plasma membrane of cells. Using the well-studied β_2_ adrenergic receptor (β_2_AR) as a prototypical GPCR^17^, we validated the utility of this platform by demonstrating that membrane-delivered β_2_AR responds to ligand binding and triggers cAMP production to rescue the wound healing defects of β_1_/β_2_AR double knockout primary cells. We expect this platform to be readily adapted to the cell-free production and nanodelivery of a broad range of GPCRs and other membrane proteins for DNA and RNA-free manipulation of cellular functions.

## Full-length production of β_2_AR-NLPs by cell-free, co-translational approaches

**Figure 1:**
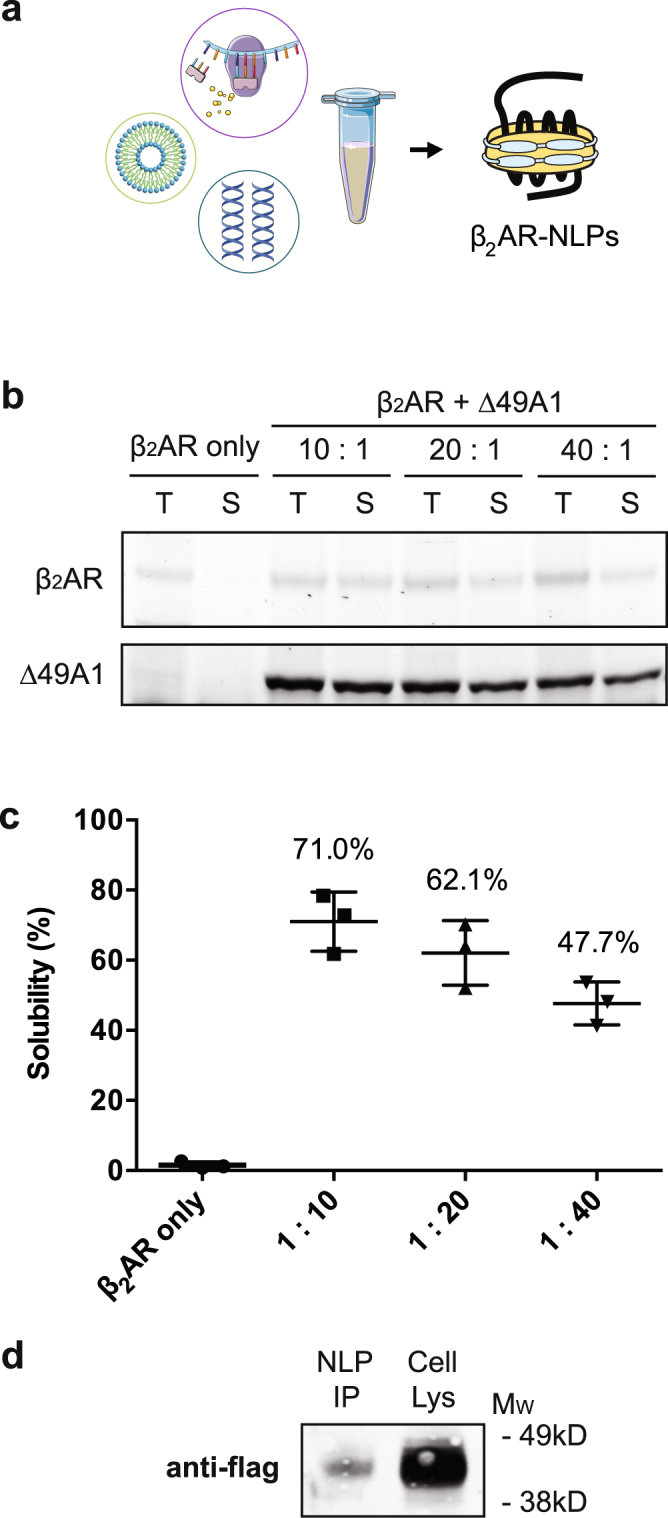
Cell-free production of β_2_AR-NLPs. (**a**) Co-translational production of β_2_AR-NLPs was achieved by incubating β_2_AR and ApoAI DNAs in the presence of synthetic lipid nanoparticles (1,2-dimyristoyl-sn-glycero-3-phosphocholine, DMPC) with the cell-free reaction mixture at 30 °C for 18 hours. (**b**) Optimization of the GPCR-NLPs *in vitro* translation system. β_2_AR was cell-free produced in the absence of Δ49A1 cDNA and titrated in the presence of Δ49A1 cDNA (10:1, 20:1, 40:1). FluoroTect™ GreenLys (Promega) was added for visualizing newly synthesized proteins. Cell-free reactions were centrifuged at 10,000 rpm for 10 minutes. 5 μl aliquots of sample before centrifugation (total fraction, T) and of the supernatant (soluble fraction, S) were used for SDS-PAGE. Gel images were taken using Molecular Imager® Gel Doc™ XR System from Biorad. (**c**) Band intensity ratio of the soluble vs. total cell-free produced β_2_AR was utilized to determine solubility. Results are shown as mean ± S.E. (**d**) The identity and molecular weight of cell-free expressed β_2_AR was verified by side-by-side comparison with cell-expressed β_2_AR, via western blot analysis using anti-flag labeling.

To achieve large-scale, cell-free expression of functional β_2_AR, we first codon-optimized the cDNA encoding human β_2_AR protein for expression using Expressway™ Maxi Cell-Free *E. coli* Expression System (Supplementary Fig. 1). To induce spontaneous insertion of β_2_AR into Δ49A1-induced NLPs, we co-expressed codon-optimized β_2_AR with human apolipoprotein A-1 lacking the amino-terminal 49 amino acids (Δ49A1) in the presence of 1,2-ditetradecanoyl-sn-glycero-3-phosphocholine (DMPC) (Fig. 1a), as previously described^11,18^.

We then maximized the production of NLP-solubilized full-length β_2_AR protein by varying the ratio of pβ_2_AR to pΔ49A1 plasmids in the cell-free co-expression reaction mix (Fig. 1b). We found that a DNA ratio of 10:1 for pβ_2_AR to p∆49A1 promoted about 70% solubilization of β_2_AR in the total translated protein fraction (Fig. 1c and Supplementary Fig. 2a). Using western blot analysis, we further confirmed the expression of full-length β_2_AR (~47 kDa) using an anti-flag antibody (Fig. 1d and Supplementary Fig. 2b).

**Figure 2:**
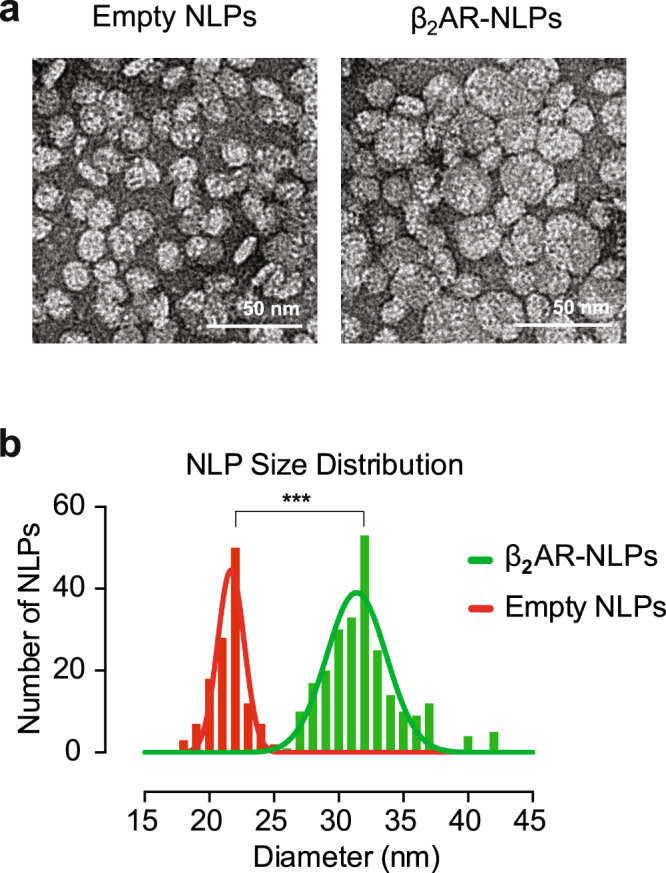
Transmission Electron Microscopy (TEM) characterization of β_2_AR-NLPs. TEM images of stain-embedded empty NLPs with average diameter of 21.5 nm and β_2_AR-NLPs with average diameter of 23 nm. Scale bar: 50 nm. (c) Particle size distribution of empty and β_2_AR-NLPs from TEM data. Standard deviation for empty and beta-adrenergic NLPs are 1.4 and 3.0, respectively (p < 0.0001, paired t test).

To conclusively demonstrate β_2_AR association with NLPs, we first purified particles by affinity chromatography via the Δ49A1 6XHis tag, followed by characterization using size-exclusion chromatography and transmission electron microscopy (TEM). The chromatographic analysis showed two clearly distinct elution peaks representing β_2_AR integrated into NLPs and empty NLPs (Supplementary Fig. 3), suggesting stable association of β_2_AR with NLPs. TEM further confirmed that approximately 27.5% of the NLPs in the purified sample contained integrated β_2_AR (Fig. 2a,b). β_2_AR-integrated NLPs displayed a significantly larger diameter than empty NLPs when imaged by TEM (β_2_AR-NLPs: 33.0 ± 3.0, empty NLP: 21.5 ± 1.4, *P* = 1.8641E-15). These results indicate that cell-free expressed β_2_AR successfully integrates into the NLP-supported bilayer.

## The **β**_2_AR-NLP complex is functional ***in vitro***

**Figure 3:**
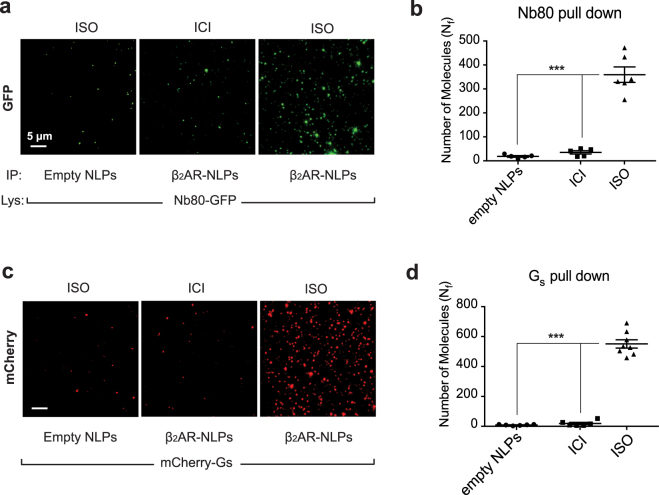
Functional characterization of β_2_AR-NLPs for binding to Nb80-GFP or cognate Gs-protein. (**a**,**c**) Purified β_2_AR-NLPs or empty NLPs were immobilized on a chip via anti-flag antibody (M2, Sigma). Nb80-GFP or Gs-mcherry-containing HEK cell lysates were flown over the immobilized β_2_AR in the presence of either the β_2_AR agonist isoproterenol (ISO, 10 μM) or the β_2_AR antagonist ICI118551 (ICI, 10 μM). (**b**,**d**) Average number of fluorescent molecules per imaging area, N_f_. Results are shown as mean ± s.d. (n > 5; ***p < 0.0001).

We next sought to investigate the functional integrity of NLP-bound β_2_AR. To do so we assayed the ligand-induced conformational changes of NLP-bound β_2_AR using a single-molecule pull-down assay (SiMPull)^19,20^, tapping into a β_2_AR conformational biosensor (Nb80-GFP) that specifically stabilizes the active conformation of β_2_AR^21^. Immobilized β_2_AR-NLPs or empty NLPs were first pre-incubated with either antagonist ICI118551 (ICI, 10 μM) or agonist isoproterenol (ISO, 10 μM) before pulling down purified Nb80-GFP. Only in the β_2_AR-NLP sample in the presence of the agonist we could detect green fluorescent spots, representing β_2_AR-bound Nb80-GFP (~313 molecules). We observed a small number of pull-down Nb80-GFP either in the presence of ICI (~31 molecules) or under the conditions when empty NLPs were immobilized (~17 molecules) (Fig. 3a,b). As an additional confirmation of the specificity of binding, we also pulled down mCherry-labeled Gs protein in the presence of ISO, but not ICI (Fig. 3c,d). Importantly, we observed different association rates of Nb80-β_2_AR binding in the presence of ligands with different pharmacological properties (Supplementary Fig. 4a). The association rates for two full agonists (ISO and epinephrine, EPI, 10 μM) and one partial agonist (dobutamine, DOB, 10 μM) are 5.3*10^8^, 1.4*10^9^ and 1.8*10^8^ M^−1^ min^−1^, respectively, which correlate with the known efficacy of the drugs examined (Supplementary Fig. 4b)^22^. These results suggest that cell-free expressed β_2_AR maintains functional integrity when integrated into NLPs and can distinguish the pharmacological properties of different ligands.

Because our TEM data allows us to determine only the percentage of NLPs-bound β_2_AR versus empty NLPs, we further evaluated the fraction of functional receptors out of the NLP-bound β_2_AR pool using SiMPull (Supplementary Fig. 5a). To do so, we compared the number of pulled down Nb80-GFP molecules in the presence of 10 μM ISO to the total number of immobilized β_2_AR-NLPs, visualized with an anti-6xHis-555 antibody (Supplementary Fig. 5b,c). Assuming that 75% GFP is fluorescent, as previously reported^20,23^, we estimated that the percentage of functional β_2_AR particles was 10% of the total purified sample, which corresponds to 30 ng (330 fmol; 2*10^11^ molecules) per microliter (ul) cell-free reaction.

## Functional insertion of cell-free expressed **β**_2_AR-NLPs in living cells

**Figure 4:**
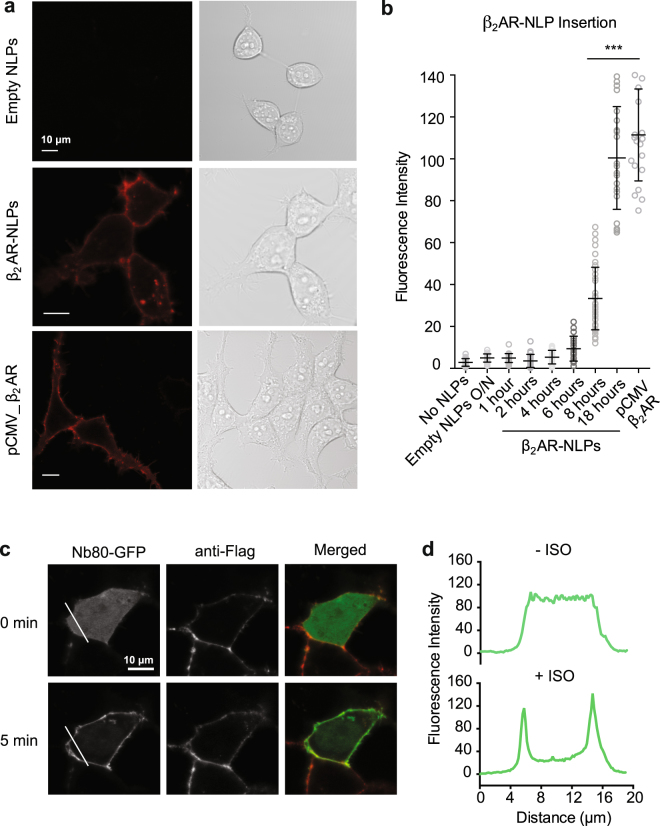
Nanodelivery of *in vitro* translated β_2_AR on cells. Time-course of β_2_AR-NLPs insertion onto HEK293 cell membranes was performed by live-cell anti-flag staining at various time points of incubation with β_2_AR-NLPs. (**a**) Representative fluorescence images showing flag staining intensity after 18 hours incubation. (**b**) Analysis of flag cell staining indicates efficient membrane insertion after overnight incubation (18 hours) (n = 20 cells, p < 0.0001). Results are shown as mean ± S.E. (**c**) Isoproterenol induced membrane-relocalization of Nb80-GFP in HEK293 cells after β_2_AR nanodelivery (18 hours, n = 8 cells). (**d**) Representative traces of fluorescence from line indicated in **c**.

Recent studies have described the application of NLPs to both *in vitro* and *in vivo* delivery of hydrophobic drugs and membrane antigens^5,6,8^. Thus, we tested whether cell-free expressed β_2_ARs could be delivered via NLPs onto cell membranes and maintain their function. When DiO-labeled empty NLPs were incubated with HEK 293 cells in serum-free medium, green fluorescence could be readily detected on the cell membrane after 14 hr incubation, suggesting efficient transfer of the lipid component of NLPs onto cell membranes (Supplementary Fig. 6a). To test nanodelivery of β_2_AR, we applied purified NLP-bound β_2_AR onto 293 cells (100 nM) for different amounts of time, followed by live-cell staining and imaging with an anti-flag antibody conjugated to Alexa-546 (Fig. 4a). After just 6 hr of incubation, red fluorescence intensity could already be detected at the cell membrane, indicating β_2_AR incorporation onto the cell membrane and continued to increase up to 18 hr of incubation, when it reached >10-fold higher intensity compared to the 6 hr timepoint (Fig. 4b).

The conformational biosensor Nb80-GFP has been previously used for direct visualization of β_2_AR activation in living cells^21^. We thus used this approach to verify that nanodelivered β_2_AR maintained functional integrity as demonstrated by efficient cytosol-to-membrane recruitment of Nb80 and production of cyclic AMP (cAMP) upon agonist exposure (Fig. 4c). After 18 h of β_2_AR nanodelivery on 293 cells expressing Nb80-GFP, we performed dual-color, time-lapse imaging of flag staining and Nb80 relocalization in response to 10 μM ISO. We observed the rapid relocalization of Nb80-GFP from the cytoplasm to the membrane, confirmed by a marked increase in green fluorescence intensity on the cell membrane (~75%), while the red fluorescence intensity on the membrane remained constant (Fig. 4d and Supplementary Fig. 6b).

**Figure 5:**
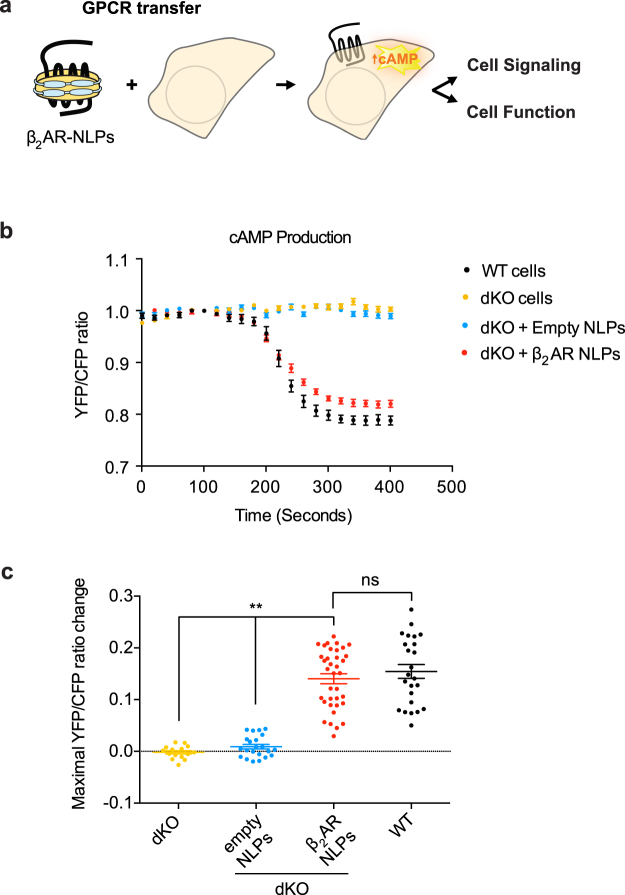
Nanodelivered β_2_AR can drive cellular signaling. (**a**) Schematic of GPCR nanodelivery to cellular membranes to alter cellular properties. (**b**,**c**) ICUE measurement of cAMP production in primary β_1_/β_2_AR double-knockout neonatal cardiac myocytes (dKO myocytes) shows that nanodisc-delivered β_2_AR is capable to elicit cellular signaling in response to the agonist isoproterenol at a level comparable to endogenous β_2_AR (**p < 0.01, unpaired t test). Results are shown as mean ± S.E.

These results suggest that cell-free–produced β_2_AR can be functionally delivered to the cell membrane, resulting in a conformational response to agonist stimulation. More importantly, when stimulated by agonist, nanodelivered β_2_AR triggered a downstream signaling cascade comparable to that of endogenous β_2_AR receptors (Fig. 5a). We measured cAMP production with the fluorescent resonance energy transfer (FRET)-based cAMP biosensor ICUE3 in living cells^24^. As isoproterenol is a nonselective β_1_AR/β_2_AR agonist and most cell types endogenously express βARs, we isolated primary neonatal cardiac myocytes from both β_1_/β_2_AR double-knockout mice (dKO) and wild-type mice. Upon isoproterenol application, we observed cAMP production demonstrated by 15% FRET changes only in wild-type, but not in dKO myocytes (Fig. 5b). When β_2_AR was nanodelivered onto dKO primary myocytes for 14 hr, we observed 14% FRET changes in response to ISO, which was similar to that observed in wild-type cells. In contrast, 14 hr incubation of dKO myocytes with empty NLPs failed to generate any detectable FRET changes (Fig. 5c).

## Receptor nanodelivery alters cellular phenotype

**Figure 6:**
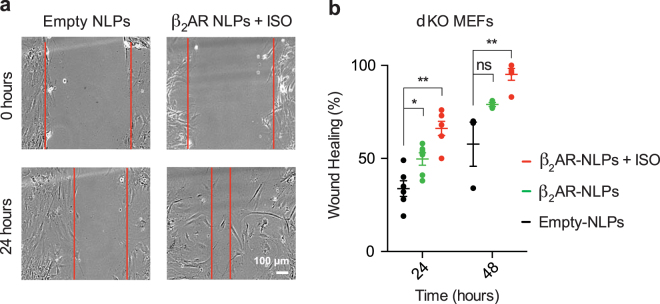
Nanodelivered β_2_AR increases rescues wound healing defects of β_1_/β_2_AR dKO mouse embryonic fibroblasts (MEFs). (**a**) Representative images of dKO MEFs treated with β_2_AR-NLPs or empty NLPs with or without isoproterenol and subjected to scratch assay for cell migration. Wound healing was analyzed at 24 and 48 hours. Red lines represent the cell front. (**b**) Wound healing was quantified as percentage of gap closure. Results are shown as mean ± S.E. (*p < 0.05, **p < 0.01, n = 3, unpaired t test).

The β_2_AR signaling has been directly linked to fibroblast migration during wound-healing processes^25,26^. Thus, we next asked whether β_2_AR nanodelivery could be used to induce a pro-migratory phenotype in cultured fibroblasts. To do so, we nanodelivered β_2_AR onto mouse embryonic fibroblasts (MEFs) from our dKO mice and then assayed their wound healing properties using an *in vitro* scratch assay. At 24 and 48 hr after scratch application, dKO MEFs displayed 33% and 57% gap closure, respectively. Upon β_2_AR nanodelivery, both with and without agonist stimulation (10 μM ISO), the wound-healing properties of these cells were significantly increased (Fig. 6a). β_2_AR-insertion without agonist application resulted in 50% and 79% gap closure at the two timepoints, indicating that basal activity of the receptor is sufficient to elicit a significant wound-healing effect (p = 0.014 after 24 hr). Agonist stimulation in addition to β_2_AR nanodelivery led to a further increase in gap closure, reaching 66% and 95% at the 24 and 48 hr time points, respectively (Fig. 6b, p = 0.0002 after 24 hr and p = 0.008 after 48 hr, n = 3). These results validate the feasibility of GPCR nanodelivery and prove that this is a suitable strategy to selectively modulate cellular signaling and phenotype.

## Discussion

In summary, we demonstrated for the first time a method for nanodelivery of membrane proteins, in which an *in vitro* one-step, cell-free system can be used to synthesize functional nanodisc-solubilized GPCRs that can be further delivered onto plasma membranes of cells to trigger signaling cascades and alter cellular function. To demonstrate the feasibility of GPCR nanodelivery and provide proof of concept for the therapeutic potential of this new delivery system, we cell-free produced nanodisc-solubilized β_2_AR and fully characterized its function both prior and after delivery on cell membranes. We validate nanodelivery of β_2_AR in three different cell types, including hard to transfect primary cells, where we show that nanodelievered β_2_AR triggers intracellular cAMP production in a similar manner to naturally occurring endogenous β_2_AR and imparts novel functional properties to the receiving cells, as evidenced by increased cell migration in an *ex vivo* wound-healing model using β_1_/β_2_AR double knockout cells.

Our method greatly expands the existing toolbox for direct protein transduction to living cells independent of DNA or RNA-based techniques^2^ and affords the benefits of cell-free, large-scale, *in vitro* protein expression (e.g., speed, purity, lower costs and no need for detergents).

Since a decade ago when membrane protein synthesis in cell-free systems started^27^, the yield-on-cost ratio has been continuously increasing. Membrane protein synthesis in cell-free systems (e.g. *E. coli* and wheat germ based cell-free systems) has started to reach levels comparable to soluble proteins^15,28^, especially with the development of a continuous exchange cell-free dialysis system (CECF) that provides a prolonged reaction time and freshly supplied reaction components^14^. Though the CECF reaction is approximately 10 times more expensive compared to a typical batch reaction, with the increased productivity, it is possible to achieve a significant reduction of the total costs by a factor of 10. The main costs of cell-free synthesis systems arise through the addition of T7 RNA polymerase, energy in the form of adenosine and guanosine triphosphate and an energy regeneration system. The costs of exogenous added T7 RNA polymerase might be decreased by using in house production. The development of alternative energy-rich components and the energy regeneration systems is already in process^16^. The yield-on-cost ratio of cell-free system for membrane protein synthesis will continue to increase given the increasing productivity of cell free systems in combination with the reduced costs for lysates and the energy regeneration system.

In the current study, we adopted an innovative approach to estimate the amount of functional GPCR product in the final expression product, which takes advantage of a protein binding assay (SiMPull). Because of the intrinsically different experimental procedures used to determine the yield of functional GPCR between this and previous studies, an exact comparison among yields produced by the different systems would not be accurate. However, the relatively overall lower efficiency of functional GPCR expression using *in vitro* translation (ng per ul cell-free reaction) compared to the broadly used insect cell-baculorivus expression system (<1 mg/L total purified proteins)^28,29^ is possibly due to the intrinsic limitations of the system. In fact, the tRNAs, amino acids, ribosomes and other components of the mixture are limited in an *in vitro* translation reaction, which further limits the time length of reaction efficacy. In addition, selection of different lipid types may further increase the solubility of the protein, though extensive characterization has been performed in the past^15,28^. Successful strategies to further optimize cell-free synthesis of GPCRs and membrane proteins in general have been done on a variety of lipid structures like liposomes, micelles, bicelles and nanodiscs^27,30,31^. Future work will focus on further increase the yields by applying modifications to the cell-free reaction conditions, such as integrating continuous exchange cell-free dialysis system and systematic optimization in selection of lipid types.

Given the efficiency of membrane protein incorporation strictly depends on the concentration of nanodisc-solubilized proteins applied onto the cells, our system also provides a much higher degree of precision in fine-tuning the membrane levels of delivered receptors than those achieved by DNA expression. The high simplicity and flexibility of this system may allow nanodelivery of a broad range of membrane proteins, such as other GPCRs, optogenetic actuators and reporters in various cell lines. Furthermore, It is promising that this system may allow nanodelivery of GPCRs to living organisms for both research and clinical uses, such as revision restoration^32,33^. However, extensive research focusing on substantial optimization, such as *in vivo* stability of the receptor complex, the bioavailability in certain tissue types and the specificity of the nanodelivery, is necessary to realize the full potential of this system for *in vivo* applications.

## Methods

### Cell-free, *in vitro* translation reaction for GPCR-NLP self-assembly

Cell-free reactions using the Expressway^TM^ Maxi Cell-Free *E. coli* Expression System (Life Technologies) were carried out as previously described^18^. The gene for β_2_AR was codon-optimized for *E. coli* expression. A Flag tag was positioned after the start codon and NdeI and SmaI restriction sites were positioned at the beginning and the end of the construct, respectively. The sequence was ordered as a Geneblock (Integrated DNA Technologies) and cloned using the NdeI-SmaI sites into pIVEX2.3d and pIVEX2.4d (Roche). The pIVEX2.3d-β_2_AR construct was used to prepare β_2_AR-NLPs for cell-membrane insertion, followed by live staining, which required the flag tag to be exposed at the very beginning of the receptor N-terminus; the pIVEX2.4d-β_2_AR construct resulted in a protein product with an N-terminal 6xHis tag preceding the flag tag and was used for all other applications. The base changes that were made during codon optimization are shown in Supplementary Fig. 3. Small, unilamellar vesicles of DMPC (liposomes) were prepared by sonication of a 25 mg/ml water–DMPC solution on ice until optical clarity was achieved (usually 10 min), followed by 2 min of centrifugation at 14,000 rcf to remove metal contamination from the sonication probe tip. DMPC small, unilamellar vesicles were added to the cell-free reaction mixture prior to starting the reaction at a final concentration of 2 mg/ml. Addition of FluoroTect™ GreenLys (Promega) was done at the beginning of the reactions to facilitate visualization and quantification of synthesized proteins. For membrane protein and NLP coexpression in a 200 μl reaction, 2.5 μg of plasmid DNAs were added to the lysate mixture. The ratio of β_2_AR to Δ49A1 plasmid DNA (containg a N-terminal HIS tag) was kept constant at 20:1. The reactions were incubated at 30 °C for 18 h. Empty NLPs were generated by omitting the β_2_AR plasmid DNA.

### Affinity purification of cell-free–produced NLPs

Empty NLPs or β_2_AR-NLPs, both containing 6xHIS tags on their ApoAI component, were purified from their respective reaction mixtures by Ni/NTA affinity purification. Briefly, we used immobilized metal ion affinity chromatography (IMAC) tips on an automatic pipette (80 μl resin volume, Mettler-Toledo). The resin was first equilibrated with native buffer (Imidazole 20 mM, NaCl 300 mM, NaHPO_4_ 50 mM, pH 8.0), then incubated with the NLP-containing reaction mixture and washed three times before elution of the NLPs in native buffer with 400 mM imidazole. Eluted NLPs were then dialyzed in 2 L of phosphate-buffered saline (PBS) buffer using 3.5 KDa molecular weight cutoff D-tube dialyzers (Millipore). Total protein concentration was measured by bicinchoninic acid (BCA) assay (Thermo) on a Synergy2 plate reader (BioTek), using a standard curve with known concentrations of BSA. SDS-PAGE and fluorescence imaging of the gels were performed as previously described^18^.

### Nb80-GFP quantification

Recombinant enhanced green fluorescent protein (EGFP; 1 mg/ml) was purchased from Vector Laboratories. Nb80-GFP containing 5xHis-tag was expressed in HEK293T cells and purified using IMAC resin tips (Rainin). A series of dilutions of EGFP and Nb80-GFP was made using Tris-EDTA buffer (10 mM Tris-HCl, 10 mM EDTA, pH 8.0) as the diluent. Fluorescence was determined by a Synergy 2 fluorescence microplate reader (BioTek Instruments) using a 485 nm, 20 nm bandpass excitation filter and a 528 nm, 20 nm bandpass emission filter with an instrument sensitivity setting of 50. Quantitation of EGFP was determined using a calibration curve determined by linear regression (r2 = 0.998).

### Transmission electron microscopy

Empty NLPs and β_2_AR-NLPs were diluted to a final concentration of 0.2 mg/mL. A deep-staining approach was utilized for sample embedding immediately prior to examination with a JEOL 1230 TEM. All samples were mixed with 16% ammonium molybdate and 0.1% trehalose and immediately transferred to a carbon-coated copper grid. The grids were further prepared for imaging with a single wash using PBS buffer and were air-dried. All TEM micrographs were captured at 60,000x magnification and processed for size distribution measurements in FIJI package. From 8 micrographs, 140 particles were measured by the long axis. The measurements for empty and β_2_AR-NLPs were plotted and standard deviations were calculated using Microsoft Excel.

### Single-molecule pull-down assay (SiMPull)

For the β_2_AR functionality test, cells were plated onto 100 mm petri dishes (VWR) at a density of 200,000 per dish and grown for 24 h before being transfected with 3 μg of Gs-mCherry plasmid DNA. Cell lysates were prepared by cell titration in apyrase reaction buffer (NEB) supplemented with protease inhibitors cocktail (Sigma) (20 mM MES, 50 mM NaCl, 5 mM CaCl_2_, 1 mM DTT, 0.05% Tween-20, pH 6.5). Complete cell lysis was ensured by probe-sonicating on ice for 30 s. Cell debris was removed by 10 min of centrifugation at 14,000 rcf (4 °C). Nearly all guanine nucleotide triphosphates and diphosphates (NTPs and NDPs) were hydrolyzed to monophosphates (GMPs) by incubating the lysate with apyrase at 30 °C for 1 h. SiMPull chips were first washed twice with T50 buffer (50 mM NaCl, 10 mM Tris-HCl, pH 7.8). Next, NeutrAvidin (Thermo) was added and slides were incubated for 5 min at room temperature (RT). After washing twice with T50, biotinylated anti-flag antibody (10 nM) was added and incubated for 10 min at RT, then washed twice with T50 again. Subsequently, 1 μg of purified cell-free–produced NLPs (either containing flag-tagged β_2_AR or empty nanodiscs as a negative control) was added, followed by 10 min incubation at RT. Unbound samples were removed from the chamber by washing twice with T50 buffer. Cell lysates expressing Nb80-GFP or Gs-mCherry were 1:10 diluted into T50 buffer containing 10 μM isoproterenol (Sigma) or ICI (Sigma), then added to the SiMPull chamber and incubated for 10 min at RT, followed by washing twice with T50 with isoproterenol or ICI. Proteins immobilized on the slides were visualized using a prism-based, total internal reflection fluorescence microscope equipped with excitation laser 488 nm (GFP) and 561 nm (mCherry) and DV2 dichroic 565dcxr dual-view emission filters (520/30 nm and 630/50 nm). Mean spot counts per image and standard deviations were calculated from images taken from 5 to 10 different regions using a script written in Matlab. For drug-specific Nb80 to β_2_AR ON rate determination: 1 μg of cell-free–produced β_2_AR-NLP was immobilized on SiMPull chips and preincubated with 10 nM drug (isoproterenol, dobutamine, or epinephrine) for 15 min. Immediately after injection of 1 nM purified Nb80 (diluted in T50 buffer containing 10 nM drug), several images were taken at each time point (0, 30 s and 1, 2, 3, 4, 5, 10, 15, 20 and 25 min). τ_1/2_ was determined by fitting the data with a one-phase association curve (GraphPad Prism 6).

### Cell culture, DNA constructs and transfection

HEK293 cells (ATCC #CRL-1573) were grown in Dulbecco’s modified Eagle’s medium (DMEM, Invitrogen) supplemented with 10% fetal bovine serum and 1 mg/ml penicillin-streptomycin. The pEGFP_N1 plasmid containing Nb80-GFP was a gift from Dr. Huang Bo (UCSF). The original rat Gs protein pcDNA was from Addgene (55793). The pcDNA plasmid containing Gs-mCherry was made by restriction cloning using a single BamHI site inserted after amino acid 71 in the Gs protein. The mCherry insert was amplified by PCR with the addition of two BamHI sites and two flexible linker regions (GGGS) on each side prior to restriction cloning. The correct insertion of mCherry was confirmed by sequencing. Cell transfections were performed with Effectene^®^ Transfection Reagent (Qiagen) according to manufacturer’s protocol. All cell-culture reagents were from Life Technologies, unless otherwise noted.

### Live-cell confocal imaging

For insertion of NLPs onto cell membranes, the cells were plated onto 35 mm glass bottom microwell dishes (MatTek) at a density of 10,000 per well and transfected with 100 ng of Nb80-GFP plasmid DNA. After 14 h, the medium was switched to serum-free medium with the addition of 10 μg of purified total NLPs (corresponding to a final concentration of approximately 55 nM). The removal of serum is meant to prevent absorption of NLPs onto serum albumin and increase the efficiency of NLP insertion onto cell membranes. To maximize the insertion onto cell membranes, cells were incubated with the NLPs overnight. For surface-labeling of cells with membrane-inserted receptors, cells were washed three times in HBSS (Life Technologies) and supplemented with 2 mM Ca^2+^ and 15 mM HEPES (pH 7.4), prior to being incubated at 37 °C for 15 min with M1 anti-flag antibody (1:1000, Sigma) conjugated to Alexa 546 (A-20002, Thermo Fisher). The cells were imaged in HBSS buffer with a 40 × oil-based objective on an inverted confocal microscope (Observer, Zeiss). Images were acquired with a CCD camera driven by the Zen software (Zeiss). Isoproterenol (Sigma) was carefully added directly onto the dish during the imaging session (final concentration 10 μM). Time-lapse images were analyzed on Image J and Nb80-GFP membrane relocalization was confirmed by drawing a line across the cell membrane and using the plot profile function. The analysis of green and red fluorescent signal change (membrane ΔF/F_0_) was done using a custom-made script written in Matlab. Briefly, the green and red fluorescence intensities were measured in a mask generated using the signal from the red channel, indicating the membrane location of flag-β_2_AR. Fluorescent signal change for each wavelength was calculated as (F-F_0_)/F_0_, with F_0_ being the fluorescence intensity prior to agonist stimulation.

### Fluorescent resonance energy transfer (FRET) measurement

Wild-type or β_1_/β_2_AR dKO myocytes were infected with adenovirus overnight for expression of the cAMP biosensor ICUE3^24^. dKO myocytes were further incubated with β_2_AR-NLPs or empty NLPs from 6 to 24 h before cells were used in FRET recording. Two adenoviruses expressing ICUE3 and flag-β_2_AR were used to co-infect dKO cells overnight, for the cell-expressed β_2_AR data. Cells were imaged on a Zeiss Axiovert 200 M microscope with a 40×/1.3NA oil-immersion objective lens and cooled CCD camera. Dual emission ratio imaging was acquired with a 420DF20 excitation filter, a 450DRLP dichroic mirror and two emission filters (475DF40 for cyan and 535DF25 for yellow). The acquisition was set with 200 ms exposure in both channels. Images in both channels were subjected to background subtraction and ratios of yellow-to-cyan color were calculated at different time points.

### Scratch assay

β_1_/β_2_AR dKO MEFs were plated onto 6-well plates in DMEM medium containing 10% FBS and incubated at 37 °C to create a confluent monolayer. β_2_AR-NLPs or empty NLPs were added to the cells at 20 nM concentration and incubated for 8 h. The cell monolayer was scraped in a straight line with a pipet tip to create a gap. The debris was removed and the cells were washed once to smooth the edge of the scratch with 1 ml of the growth medium, then incubated with 4 ml of medium. Immediately following the scratch, isoproterenol or PBS vehicle was added to cells, with a final isoproterenol concentration of 10 μM. Images of the scratches were taken under a phase-contrast microscope. Additional pictures were taken at the 24- and 48-h time points. The images acquired at each time point were analyzed for percentage gap closure on ImageJ.

### Statistical analysis

To quantify GPCR insertion efficiency into NLPs from β_2_AR TEM images, a semi-automated custom-made particle-selecting tool was used. To quantify the particles based on diameter distribution, particle analysis tools from Image J were employed. The statistical distribution of NLPs revealed that both the mean and the mode for the size of GPCR-filled NLPs were 23.5 nm, with about 27% of insertion efficiency. For quantification of β_2_AR insertion on the cell membrane, statistical analisis was performed using one-way ANOVA with Dunnett’s post-test, for comparison of every condition versus control condition (Empty-NLPs). During scratch assay, on the outer bottom of the dish an ultrafine ink marker was used to lay reference points close to the scratch to facilitate retrieving the same field for all image acquisitions. For TEM nanodisc size distribution statistical analysis was performed using paired t-test. All other statistical analyses were performed using unpaired t test.

## Electronic supplementary material

Supplementary Information

## References

[1] Ghosh E, Kumari P, Jaiman D, Shukla AK (2015). Methodological advances: the unsung heroes of the GPCR structural revolution. Nat Rev Mol Cell Biol.

[2] D’Astolfo DS (2015). Efficient intracellular delivery of native proteins. Cell.

[3] Denisov IG, Sligar SG (2016). Nanodiscs for structural and functional studies of membrane proteins. Nat Struct Mol Biol.

[4] Leitz, A. J., Bayburt, T. H., Barnakov, A. N., Springer, B. A. & Sligar, S. G. Functional reconstitution of Beta2-adrenergic receptors utilizing self-assembling Nanodisc technology. *Biotechnique*s **40**, 601–602, 604, 606, passim (2006). 10.2144/000112169 16708760

[5] Fischer NO (2014). Evaluation of nanolipoprotein particles (NLPs) as an *in vivo* delivery platform. PLoS One.

[6] Ghosh M, Ryan RO (2014). ApoE enhances nanodisk-mediated curcumin delivery to glioblastoma multiforme cells. Nanomedicine (Lond).

[7] Numata M (2013). Nanodiscs as a therapeutic delivery agent: inhibition of respiratory syncytial virus infection in the lung. Int J Nanomedicine.

[8] Kuai R, Ochyl LJ, Bahjat KS, Schwendeman A, Moon JJ (2017). Designer vaccine nanodiscs for personalized cancer immunotherapy. Nat Mater.

[9] Corin K (2011). A robust and rapid method of producing soluble, stable and functional G-protein coupled receptors. PLoS One.

[10] Gao T (2012). Characterization of de novo synthesized GPCRs supported in nanolipoprotein discs. PLoS One.

[11] Yang JP, Cirico T, Katzen F, Peterson TC, Kudlicki W (2011). Cell-free synthesis of a functional G protein-coupled receptor complexed with nanometer scale bilayer discs. BMC Biotechnol.

[12] Shilling PJ, Bumbak F, Scott DJ, Bathgate RAD, Gooley PR (2017). Characterisation of a cell-free synthesised G-protein coupled receptor. Sci Rep.

[13] Orban E, Proverbio D, Haberstock S, Dotsch V, Bernhard F (2015). Cell-free expression of G-protein-coupled receptors. Methods Mol Biol.

[14] Quast RB, Sonnabend A, Stech M, Wustenhagen DA, Kubick S (2016). High-yield cell-free synthesis of human EGFR by IRES-mediated protein translation in a continuous exchange cell-free reaction format. Sci Rep.

[15] Shinoda T (2016). Cell-free methods to produce structurally intact mammalian membrane proteins. Sci Rep.

[16] Anderson MJ, Stark JC, Hodgman CE, Jewett MC (2015). Energizing eukaryotic cell-free protein synthesis with glucose metabolism. FEBS Lett.

[17] Rasmussen SG (2011). Crystal structure of the beta2 adrenergic receptor-Gs protein complex. Nature.

[18] He W (2015). Cell-free expression of functional receptor tyrosine kinases. Sci Rep.

[19] Jain A, Liu R, Xiang YK, Ha T (2012). Single-molecule pull-down for studying protein interactions. Nat Protoc.

[20] Jain A (2011). Probing cellular protein complexes using single-molecule pull-down. Nature.

[21] Irannejad R (2013). Conformational biosensors reveal GPCR signalling from endosomes. Nature.

[22] Baker JG (2010). The selectivity of beta-adrenoceptor agonists at human beta1-, beta2- and beta3-adrenoceptors. Br J Pharmacol.

[23] Ulbrich MH, Isacoff EY (2007). Subunit counting in membrane-bound proteins. Nat Methods.

[24] DiPilato LM, Zhang J (2009). The role of membrane microdomains in shaping beta2-adrenergic receptor-mediated cAMP dynamics. Mol Biosyst.

[25] Le Provost GS, Pullar C (2015). E. beta2-adrenoceptor activation modulates skin wound healing processes to reduce scarring. J Invest Dermatol.

[26] Pullar CE, Isseroff RR (2006). The beta 2-adrenergic receptor activates pro-migratory and pro-proliferative pathways in dermal fibroblasts via divergent mechanisms. J Cell Sci.

[27] Kalmbach R (2007). Functional cell-free synthesis of a seven helix membrane protein: *in situ* insertion of bacteriorhodopsin into liposomes. J Mol Biol.

[28] Saarenpaa T, Jaakola VP, Goldman A (2015). Baculovirus-mediated expression of GPCRs in insect cells. Methods Enzymol.

[29] Schutz M (2016). Directed evolution of G protein-coupled receptors in yeast for higher functional production in eukaryotic expression hosts. Sci Rep.

[30] Bayburt TH, Sligar SG (2010). Membrane protein assembly into Nanodiscs. FEBS Lett.

[31] Lyukmanova EN (2012). Lipid-protein nanodiscs for cell-free production of integral membrane proteins in a soluble and folded state: comparison with detergent micelles, bicelles and liposomes. Biochim Biophys Acta.

[32] Gaub BM, Berry MH, Holt AE, Isacoff EY, Flannery JG (2015). Optogenetic Vision Restoration Using Rhodopsin for Enhanced Sensitivity. Mol Ther.

[33] Gaub BM (2014). Restoration of visual function by expression of a light-gated mammalian ion channel in retinal ganglion cells or ON-bipolar cells. Proc Natl Acad Sci USA.

