# Supplementary material for: Nanodelivery of a functional membrane receptor to manipulate cellular phenotype
Source: Sci Rep. 2018 Feb 23;8:3556. doi: 10.1038/s41598-018-21863-3 (PMC5824837; doi:10.1038/s41598-018-21863-3)
Supplement: Supplementary file 1 — Supplementary Information [file 41598_2018_21863_MOESM1_ESM.pdf]

Supplementary Information

Nanodelivery of a functional membrane receptor to manipulate cellular phenotype

Tommaso Patriarchi[1,7], Ao Shen[2,7], Wei He[3], Mo Baikoghli[4], R. Holland Cheng[4], Yang K. Xiang[2,5], Matthew A. Coleman[3,6*], and Lin Tian[1*]

[1]University of California Davis School of Medicine, Biochemistry and Molecular Medicine, Davis, California, USA.

[2]University of California Davis School of Medicine, Pharmacology, Davis, California, USA.

[3]Lawrence Livermore National Laboratory, Livermore, California, USA.

[4]University of California Davis, Department of Molecular and Cellular Biology, California, USA.

[5]Northern California Health care system, Mather, California, USA.

[6]University of California Davis School of Medicine, Radiation Oncology, Sacramento, California, USA.

[7]These authors contributed equally to this work.

* email: lintian@ucdavis.edu or coleman16@llnl.gov

Supplementary Results

DNA encoding β_2AR comparing *E. coli* codon optimized sequence to human mRNA sequence.

```
E. COLI   ATGGGTCAGCCGGGTAATGGTAGCGCTTTTCTCTTAGCACCGAATAGAAGCCATGCACCT   60
HUMAN     ATGGGGCAACCCGGGAACGGCAGCGCCTTCTTGCTGGCACCCAATAGAAGCCATGCGCCG   60
          ***** ** ** ** ** ** ***** **  *  * ***** ************** ** 

E. COLI   GATCACGATGTTACCCAACAACGTGATGAGGTCTGGGTTGTCGGCATGGGTATTGTGATG  120
HUMAN     GACCACGACGTCACGCAGCAAAGGGACGAGGTGTGGGTGGTGGGCATGGGCATCGTCATG  120
          ** ***** ** ** ** *** * ** ***** ***** ** ******** ** ** ***

E. COLI   TCCCTGATCGTGCTGGCGATTGTCTTTGGCAACGTGCTGGTTATCACGGCCATCGCCAAG  180
HUMAN     TCTCTCATCGTCCTGGCCATCGTGTTTGGCAATGTGCTGGTCATCACAGCCATTGCCAAG  180
          ** ** ***** ***** ** ** ******** ******** ***** ***** ******

E. COLI   TTTGAACGCCTGCAGACGGTTACGAACTACTTCATCACGAGCCTGGCATGTGCGGATCTG  240
HUMAN     TTCGAGCGTCTGCAGACGGTCACCAACTACTTCATCACTTCACTGGCCTGTGCTGATCTG  240
          ** ** ** *********** ** **************    ***** ***** ******

E. COLI   GTGATGGGCTTGGCAGTCGTCCCGTTTGGTGCGGCACACATTTTGATGAAAATGTGGACC  300
HUMAN     GTCATGGGCCTGGCAGTGGTGCCCTTTGGGGCCGCCCATATTCTTATGAAAATGTGGACT  300
          ** ****** ******* ** ** ***** ** ** ** *** * ************** 

E. COLI   TTTGGTAATTTCTGGTGCGAATTTTGGACGAGCATCGACGTTCTGTGCGTTACCGCCAGC  360
HUMAN     TTTGGCAACTTCTGGTGCGAGTTTTGGACTTCCATTGATGTGCTGTGCGTCACGGCCAGC  360
          ***** ** *********** ********   *** ** ** ******** ** ******

E. COLI   ATTGAGACCCTGTGTGTTATTGCGGTGGACCGCTACTTCGCGATTACCTCCCCGTTCAAA  420
HUMAN     ATTGAGACCCTGTGCGTGATCGCAGTGGATCGCTACTTTGCCATTACTTCACCTTTCAAG  420
          ************** ** ** ** ***** ******** ** ***** ** ** ***** 

E. COLI   TATCAAAGCCTGCTGACCAAGAATAAGGCACGTGTGATCATCCTGATGGTGTGGATCGTG  480
HUMAN     TACCAGAGCCTGCTGACCAAGAATAAGGCCCGGGTGATCATTCTGATGGTGTGGATTGTG  480
          ** ** *********************** ** ******** ************** ***

E. COLI   AGCGGTTTGACTAGCTTCTTGCCGATTCAGATGCATTGGTACCGTGCGACCCATCAAGAG  540
HUMAN     TCAGGCCTTACCTCCTTCTTGCCCATTCAGATGCACTGGTACCGGGCCACCCACCAGGAA  540
             **  * **   ********* *********** ******** ** ***** ** ** 

E. COLI   GCGATCAACTGCTACGCCAACGAAACTTGTTGTGATTTCTTTACCAATCAAGCGTATGCC  600
HUMAN     GCCATCAACTGCTATGCCAATGAGACCTGCTGTGACTTCTTCACGAACCAAGCCTATGCC  600
          ** *********** ***** ** ** ** ***** ***** ** ** ***** ******

E. COLI   ATTGCGAGCAGCATTGTCAGCTTCTATGTTCCACTGGTCATCATGGTGTTTGTTTATAGC  660
HUMAN     ATTGCCTCTTCCATCGTGTCCTTCTACGTTCCCCTGGTGATCATGGTCTTCGTCTACTCC  660
          *****      *** **   ****** ***** ***** ******** ** ** **   *

E. COLI   CGTGTGTTTCAAGAGGCTAAACGTCAACTGCAGAAAATCGACAAATCGGAGGGCCGTTTC  720
HUMAN     AGGGTCTTTCAGGAGGCCAAAAGGCAGCTCCAGAAGATTGACAAATCTGAGGGCCGCTTC  720
           * ** ***** ***** *** * ** ** ***** ** ******** ******** ***

E. COLI   CACGTTCAGAACCTGTCTCAGGTTGAACAGGATGGTCGCACCGGTCACGGTCTGCGTCGC  780
HUMAN     CATGTCCAGAACCTTAGCCAGGTGGAGCAGGATGGGCGGACGGGGCATGGACTCCGCAGA  780
          ** ** ********    ***** ** ******** ** ** ** ** ** ** **  * 

E. COLI   AGCTCTAAGTTTTGCCTTAAGGAACATAAGGCGCTGAAAACGCTGGGCATCATCATGGGT  840
HUMAN     TCTTCCAAGTTCTGCTTGAAGGAGCACAAAGCCCTCAAGACGTTAGGCATCATCATGGGC  840
```

```
E. COLI   AGCTCTAAGTTTTGCCTTAAGGAACATAAGGCGCTGAAAACGCTGGGCATCATCATGGGT  840
HUMAN     TCTTCCAAGTTCTGCTTGAAGGAGCACAAAGCCCTCAAGACGTTAGGCATCATCATGGGC  840
             ** ***** *** * ***** ** ** ** ** ** *** * **************

E. COLI   ACGTTCACGCTGTGCTGGCTGCCGTTTTTCATTGTCAATATCGTCCACGTGATTCAGGAT  900
HUMAN     ACTTTCACCCTCTGCTGGCTGCCCTTCTTCATCGTTAACATTGTGCATGTGATCCAGGAT  900
          ** ***** ** *********** ** ***** ** ** ** ** ** ***** ******

E. COLI   AATCTGATTCGTAAAGAAGTATACATTCTGCTGAACTGGATCGGTTATGTTAATAGCGGC  960
HUMAN     AACCTCATCCGTAAGGAAGTTTACATCCTCCTAAATTGGATAGGCTATGTCAATTCTGGT  960
          ** ** ** ***** ***** ***** ** ** ***** ** ***** ***   **

E. COLI   TTTAACCCGCTGATTTACTGTCGTTCTCCGGACTTCCGCATCGCATTCCAAGAACTGTTG 1020
HUMAN     TTCAATCCCCTTATCTACTGCCGGAGCCCAGATTTCAGGATTGCCTTCCAGGAGCTTCTG 1020
          ** ** ** ** ** ***** **    ** ** *** * ** ** ***** ** **  **

E. COLI   TGCCTGCGTCGTAGCTCCCTGAAGGCTTACGGTAATGGTTACAGCAGCAATGGCAATACC 1080
HUMAN     TGCCTGCGCAGGTCTTCTTTGAAGGCCTATGGGAATGGCTACTCCAGCAACGGCAACACA 1080
          ********  *    **  ******* ** ** ***** ***  ****** ***** **

E. COLI   GGCGAACAGTCCGGCTATCACGTTGAGCAAGAGAAAGAGAACAAGCTGCTGTGTGAGGAC 1140
HUMAN     GGGGAGCAGAGTGGATATCACGTGGAACAGGAGAAAGAAAATAAACTGCTGTGTGAAGAC 1140
          ** ** ***   ** ******** ** ** ******** ** ** *********** ***

E. COLI   TTACCGGGTACCGAGGACTTCGTGGGCCACCAGGGCACCGTCCCGAGCGACAACATTGAC 1200
HUMAN     CTCCCAGGCACGGAAGACTTTGTGGGCCATCAAGGTACTGTGCCTAGCGATAACATTGAT 1200
           * ** ** ** ** ***** ******** ** ** ** ** ** ***** ********

E. COLI   TCTCAGGGTCGCAACTGCAGCACCAACGACAGCCTGCTGTAA                   1242
HUMAN     TCACAAGGGAGGAATTGTAGTACAAATGACTCACTGCTGTAA                   1242
          ** ** **  * ** ** ** ** ** ***   *********
```

Supplementary Figure 1: Alignment of the E. Coli codon-optimized β_2AR with the human β_2AR gene transcript DNA sequence. The alignment was done using the multiple sequence alignment feature of Clustal W. The * sign indicates base identity between the two sequences.

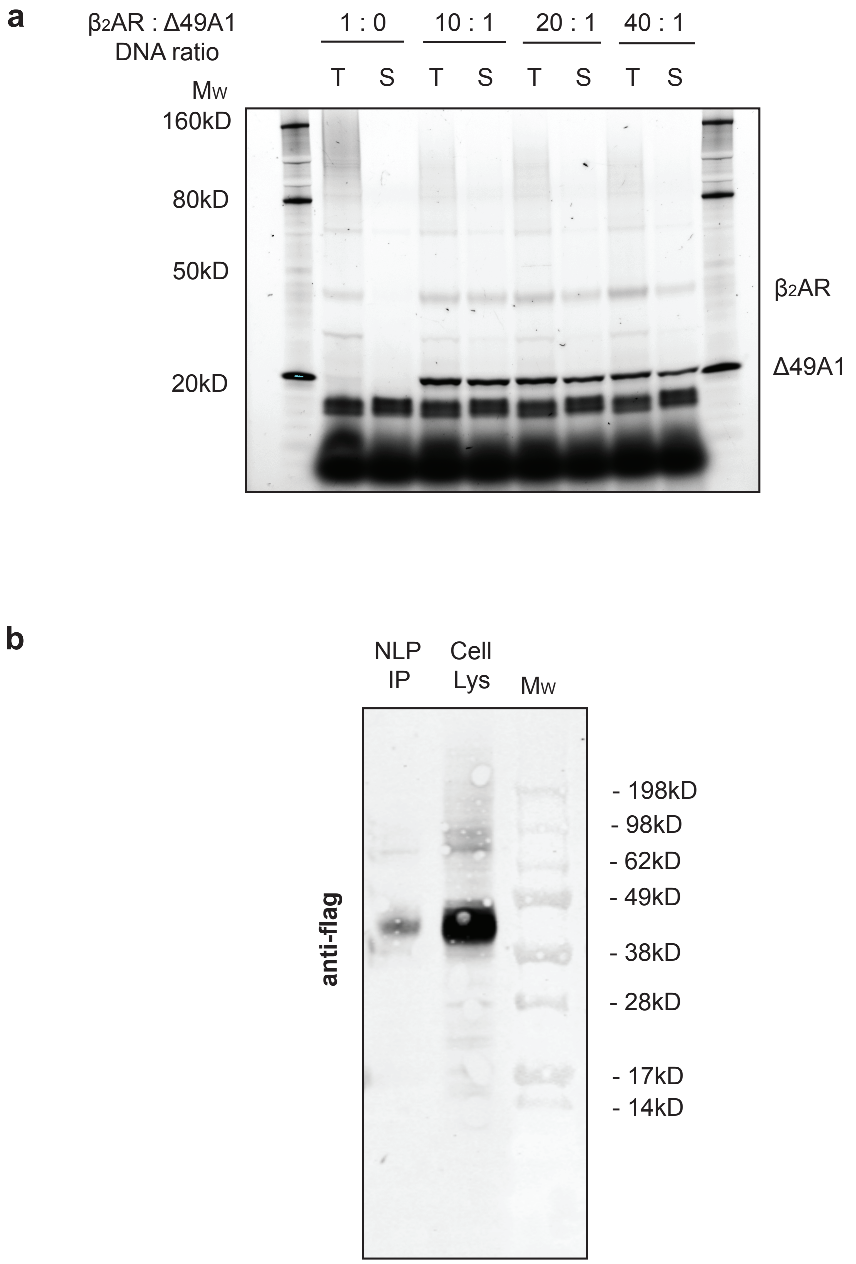

Supplementary Figure 2: Optimization of the GPCR-NLPs in vitro translation system.
(a,**b**) *Full-length versions of gel images presented in figure 1.*

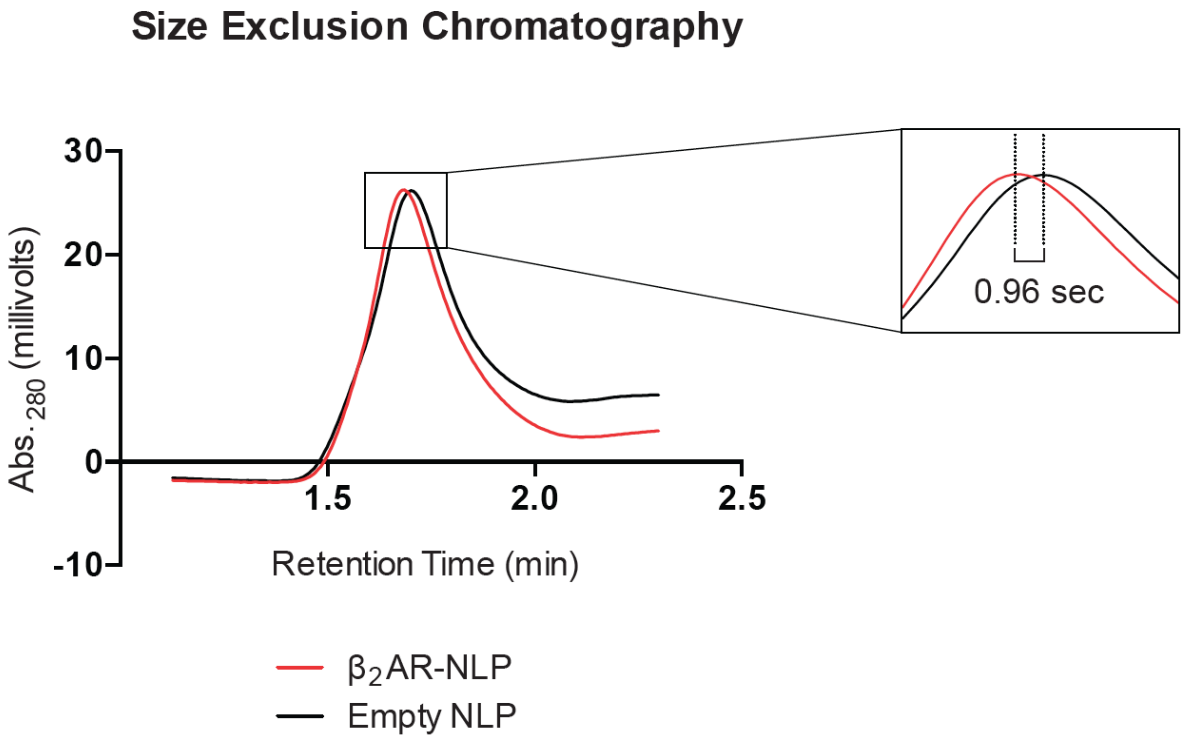

Supplementary Figure 3: Size-exclusion chromatography (SEC) characterization of β_2AR-NLPs in vitro translation product. Purified empty-NLPs and purified β_2AR-NLPs samples showed different elution times upon running onto SEC columns, indicating that the presence of the GPCR affects the overall size of the NLPs within the sample. β_2AR-NLPs and empty NLPs were analyzed by SEC (Superdex™ 200 Increase 5/150 GL, GE Healthcare, Piscataway, NJ) at a flow rate of 0.6 mL/min in PBS buffer

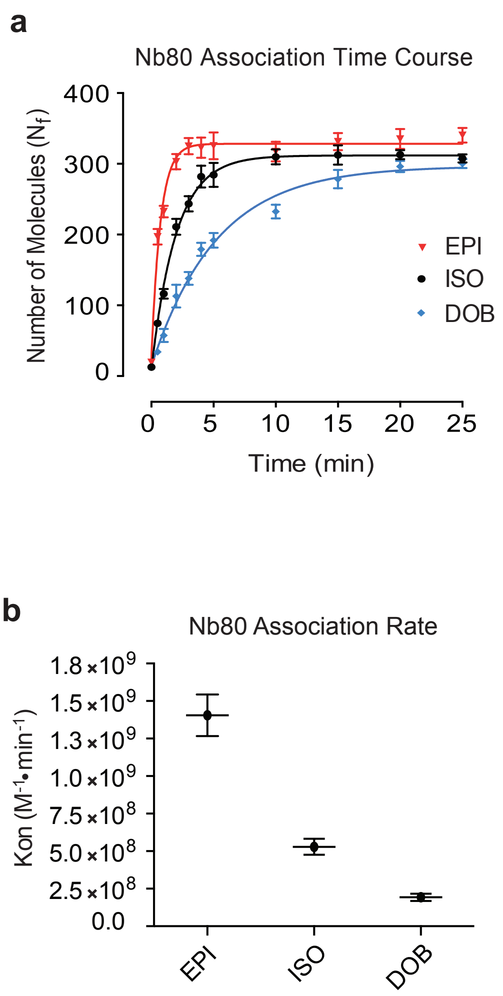

Supplementary Figure 4: Quantification of binding kinetics of Nb80-GFP to immobilized β_2AR-NLPs in SiMPull assay. (**a**) Time course of Nb80-GFP binding was obtained by measuring the amount of pulled-down molecules at different time points after initial incubation (each drug was used at 10 μM). Curves were fit with a "One step Association" curve using GraphPad Prism to obtain Kon values shown in (**b**).

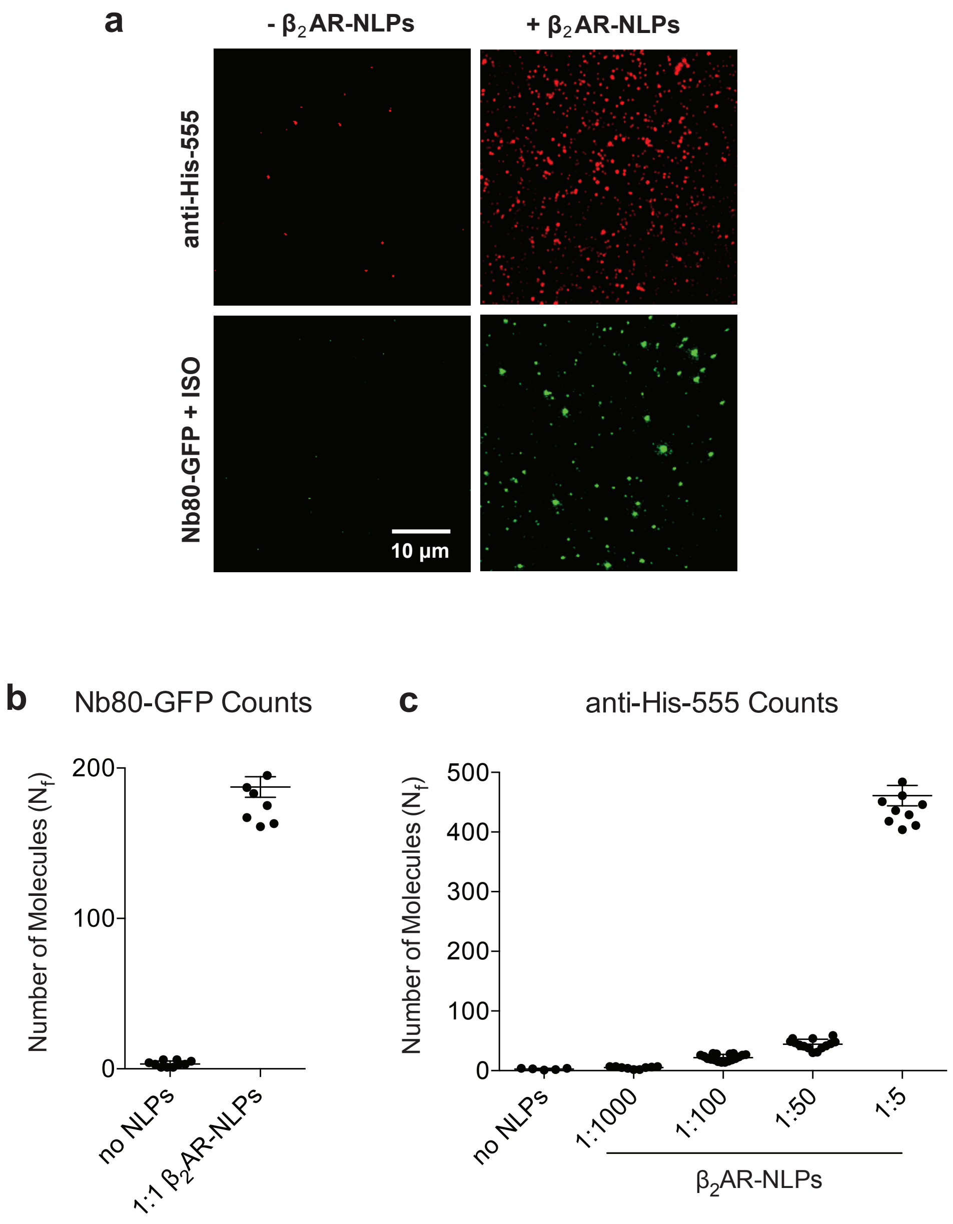

a
- β2AR-NLPs
+ β2AR-NLPs
anti-His-555
Nb80-GFP + ISO
10 µm
b
Nb80-GFP Counts
Number of Molecules (Nf)
200
100
0
no NLPs
1:1 β2AR-NLPs
c
anti-His-555 Counts
Number of Molecules (Nf)
500
400
300
200
100
0
no NLPs
1:1000
1:100
1:50
1:5
β2AR-NLPs

Supplementary Figure 5: Single-molecule quantification of functional GPCR-NLPs. (**a**) Representative images of β_2AR-NLPs immobilized on anti-Flag-M2 antibody coated SiMPull slides labeled by either 100nM Nb80-GFP plus 10uM isoproterenol (ISO) for visualizing functional β_2AR or 10nM 6xHis antibody conjugated with Alexa-Fluor 555 (anti-6xHis-555, Thermo Fisher) for visualizing total β_2AR. Specific staining was only observed from IVT samples containing 6xHis-Flag tagged β_2AR-NLPs (right panel), but not samples without β_2AR-NLPs (left panel). (**b,c**) Quantification of Nb80-GFP pull-down and total β_2AR-NLPs labeling. For anti-6xHis-555 staining, a serial dilution of the sample was used to prevent saturation of the image. The initial solution of purified β_2AR-NLPs was diluted according to the factor shown in the graph from concentration of 1.3 μg/μl. Data are represented as mean ± SEM.

a

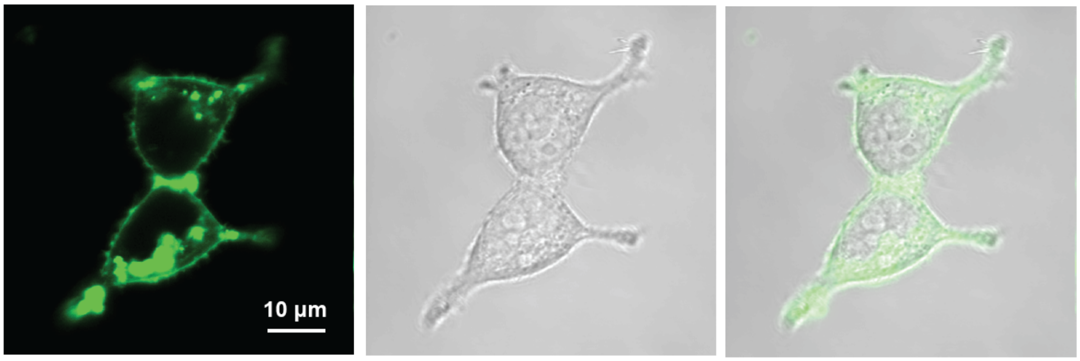

b

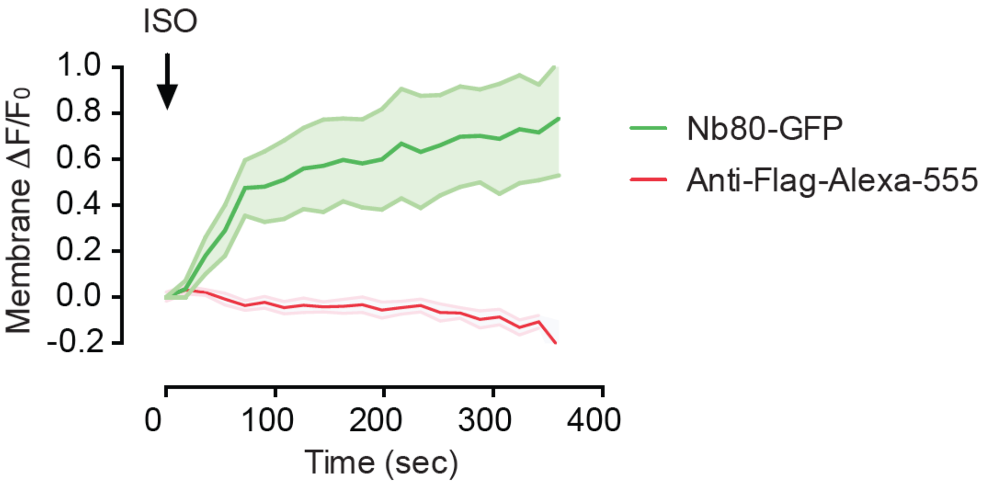

Supplementary Figure 6: (**a**) Incorporation of empty NLPs onto HEK cell membrane was monitored by labeling NLPs with a fluorescent lipophilic dye with absorption around 488 nm (DiO, Invitrogen) prior to Ni/NTA purification and delivery to the cells. Representative images of 488 nm fluorescence (left), brightfield (middle), merged (right). (**b**) Quantification of green and red fluorescence change at the membrane of Nb80-GFP-expressing HEK cells (membrane $\Delta F/F_0$) after β_2AR nanodelivery and agonist stimulation (ISO, 10 µM). Arrow indicated application of the agonist at time 0 of the curve (n=8 cells).
